# Possible protective role of 17β-estradiol against COVID-19

**DOI:** 10.46439/allergy.1.010

**Published:** 2020-08-19

**Authors:** Nabab Khan

**Affiliations:** Department of Biomedical Sciences, University of North Dakota School of Medicine and Health Sciences, Grand Forks, North Dakota 58203, USA

## Abstract

Severe acute respiratory syndrome-coronavirus 2 (SARS-CoV-2) is the virus that causes coronavirus disease 2019 (COVID-19); a worldwide pandemic as declared by the World Health Organization (WHO). SARS-CoV-2 appears to infect cells by first binding and priming its viral-spike proteins with membrane-associated angiotensin-converting enzyme 2 (ACE2) and transmembrane protease serine 2 (TMPRSS2). Through the coordinated actions of ACE2 and TMPRSS2, SARS-CoV-2 spike proteins fuse with plasma membranes and ultimately the virus enters cells. ACE2 is integral to the renin-angiotensin-aldosterone system (RAAS), and SARS-CoV-2 down-regulates protein expression levels of ACE2. Once infected, patients typically develop acute respiratory distress syndrome (ARDS) and a number of other severe complications that result in a high rate of fatality, especially in older (>60 years) adults and in people with pre-existing medical conditions. Data now indicate clearly that among people of all age groups, COVID-19 fatalities are higher in men than women. Here, attention is focused on these sex differences and posit a role of estrogen in these differences as well as possible therapeutic and protective actions of 17β-estradiol against COVID-19.

## Introduction

Severe acute respiratory syndrome coronavirus-2 (SARS-CoV-2) is a novel virus in the coronaviridae family that causes the disease now termed coronavirus 2019 (COVID-19) [1,2]. SARS-CoV-2 is a large single-stranded RNA enveloped virus [3] that reportedly originated in China [3] and that has now spread quickly worldwide leaving behind severe medical complications, high levels of fatality, and serious disruptions in normal daily living and global economies [4]. Accordingly, the WHO declared COVID-19 to be a pandemic.

SARS-CoV-2 infects cells by binding viral-spike proteins with membrane-associated angiotensin-converting enzyme 2 (ACE2) and priming of spike proteins by transmembrane protease serine 2 (TMPRSS2). Together ACE2 and TMPRSS2 facilitate the entry of SARS-CoV2 into cells by promoting the fusion of viral proteins with host plasma membranes [5,6]. The ACE2 receptor is expressed on different types of cells and tissues including human airway epithelia, lung parenchyma, vascular endothelia, kidney cells, central nervous system (CNS) cells, and small intestinal cells [7,8]. However, SARS-CoV-2 primarily infects airway epithelial cells and induces severe symptoms in infected people including acute respiratory distress syndrome (ARDS) [9,10].

ACE2, the presumed receptor for SARS-CoV-2, is involved in the regulation of the renin-angiotensin-aldosterone system (RAAS) that, among other things, catalyzes the conversion of angiotensin II to Ang 1–7. Ang-(1–7) binds to its mas receptor (MasR) that plays an important role in controlling the homeostatically regulated ACE/Ang-II/AT1 receptor axis [11–14]. ACE2 is protective against various severe pathological complications including pulmonary disease, acute respiratory distress syndrome [15–17], asthma [18,19], chronic obstructive pulmonary disease [20,21], vasoconstriction [22,23], oxidative stress [24,25], diabetes [26,27], and inflammation [28,29]. A different coronavirus, SARS-CoV, has been shown to hijack the same ACE2 receptor for viral entry into cells and SARS-CoV infection can result in acute lung injury by damaging the RAAS system [30]. Similarly, SARS-CoV-2 can cause downregulation of ACE2, damage to the RAAS system, and clinical development of ARDS [31]. ARDS is a consequence of an inflammatory storm, a key mechanism underlying the high fatality rate associated with COVID-19 [32].

Worldwide, in all age groups, more men are dying from COVID-19 than are women [33–37]. Data from New York City demonstrated that 61% of COVID-19 related deaths were men [38]. In China and Italy, COVID-19 related death rates of men were about double those of women [39]. In Australia, similar results were observed, but in their case, the cohort was restricted to people 70–85 years of age [40]. For other coronaviruses too including SARS-CoV and Middle East respiratory syndrome-coronavirus (MERS-CoV), fatalities are higher in men compared to women [41,42]. This might be related specifically to coronaviruses and not all viruses because, for example, no clear sex-based differences were observed with HIV-1 [43].

Men might be more susceptible to coronavirus-induced illnesses because of less robust immune responses [44–46]. On the other hand, women might be less susceptible because of strong innate and adaptive immune responses [47–50]. One explanation for these differences includes the presence of female hormones, which have been found to protect against infection by multiple viruses including influenza [51–53], MERS-CoV, and SARS-CoV [54]. Therefore, it is important to know the extent to which hormones and other factors such as the immune system, behavior, and genes account for the higher susceptibility rates of men versus women for COVID-19 related deaths. Accordingly, we review the literature about the possible protective roles of 17β-estradiol against COVID-19.

## Protective Actions of 17β-estradiol

17β-estradiol is a female sex hormone that plays essential roles in the development and maintenance of the female reproductive system and women’s secondary sex characteristics [55]. The physiological effects of 17β-estradiol are mediated by a family of receptors including estrogen-receptor-α (ER-α), estrogen receptor-β (ER-β), and GPR30/GPER-1 (membrane-bound G protein-coupled estrogen receptor) [56]. ER-α and ER-β are typically considered to be nuclear steroid receptors, but are in fact associated with plasma membranes, cytoplasm as well as the nucleus. Both ER-α and ER-β are involved in both cellular signaling and the regulation of gene expression through induction of ligand-activated transcription factors and direct binding to promoter-associated estrogen response elements (ERE) of target genes [57–59]. 17β-estradiol protects against multiple pathological complications including ARDS [60–64], hypertension [65,66], atherosclerosis [67], vasoconstriction [68,69], fibrosis [70,71], inflammation [72–74], autoimmune diseases [75–77], viral infections [53,78–80], and neurological disorders [81–83]. Because of such wide-ranging effects, it is important to consider the extent to which 17β-estradiol might control SARS-CoV-2 and the expression of this virus’s associated disease COVID-19 [84–88] through its ability to affect the RAAS system, anti-inflammatory and anti-viral responses, and upregulation of endolysosomal degradation pathways.

## Sex and RAAS

RAAS is a hormone/enzyme system that regulates functions of multiple organs including lungs, heart, brain, vasculature, kidneys, liver, and pancreas [89]. RAAS is composed of the classical ACE/Ang-II/AT1 axis and the non-classical ACE2/ang-(1–7)/Mas axis [90]. The renin-catalyzed conversion of angiotensin to angiotensin-I is followed by the ACE-catalyzed conversion of Ang I to Ang II; Ang II activates the AT1 (Angiotensin II type 1) receptor. The non-classical pathway consists of ACE2, Ang 1–7 (Angiotensin I-7)-Ang II receptor AT2, and the Angiotensin II receptor type 2-Mas axis. The ACE2/ang-(1–7)/Mas axis is a master regulator of the RAAS system; it controls the ACE/Ang-II/AT1R axis [90,91]. However, when dysfunctional the ACE2/ang-(1–7)/Mas axis can lead to ARDS [16,17,92], hypertension [11,91,93], and inflammation [28,94].

The RAAS system is differentially regulated in a sex-dependent manner [95–97]. Men have higher expression levels of the ACE/Ang-II/AT1R axis, whereas the ACE2/ang-(1–7)/Mas is more active in women [97,98]. Indeed, in women, treatment with 17β-estradiol enhanced the ACE2/ang-(1–7)/Mas receptor axis [95,99–102]. In contrast, testosterone was less effective than was 17β-estradiol even though testosterone has been shown to downregulate angiotensin II type 2 receptor by androgen-receptor-mediated signaling pathways [95,99,103–105]. Levels of 17β-estradiol decline with age in post-menopausal women [106] and so do activity levels of the 17β-estradiol-controlled ACE2/ang-(1–7)/Mas axis. The consequence of these age-related changes is greater susceptibility to RAAS-related pathologies [105,107–109] including ARDS and other acute lung diseases [110,111], hypertension [112], cardiovascular [113], inflammation [109,114], and fibrosis [115].

Multiple respiratory viruses including HCoV-NL-63 [116], H5N1 [117], H7N9 [118] and SARS-CoV [119] all cause acute lung injury, decrease protein expression levels of ACE2, and disturb RAAS. 17β-Estradiol increases protein expression levels of ACE2 [91,120] and suppresses lung injuries in influenza [51] and SARS-CoV [54]. Implicated in these effects are decreased inflammation and infiltration of immune cells in lungs, protection of atrial myocardia by modifying RAAS [54,102], and protection against pulmonary arterial hypertension by enhancing ACE2 [91,120]. The notion that 17β-estradiol might be protective is further supported by findings that higher expression of ACE2 protects against various factors including lipopolysaccharides, aging, and comorbid conditions like diabetes [11,121–123]; all linked to RAAS. Hence, 17β-estradiol administration could rescue the SARS-CoV-2 infection caused low levels of ACE2 remains to be further investigated.

## Sex-biased Immune Responses

Innate and adaptive immune responses control host-pathogen interactions [124,125], and women appear to have more robust immune responses than do men [44,126]; for viral infections this may be due to more efficient clearance of viruses [51,52]. However, robust immune responses in women may also lead to detrimental outcomes [126–128]. Sex-biased responses to viral infection are dependent on many factors including the presence of disease susceptible genes, different copy numbers of X-linked genes, and sex-dependent steroid hormones [47,126,128–130]. X-linked genes (eg. TLR7) and sex-specific hormones (eg. estrogen and testosterone) can affect adaptive and innate immune responses to pathogens [48–50,73,126,129]. TLR and NLR (NOD-like receptor) are known as pathogen-recognizing receptors (PRRs), which recognize diverse pathogen-associated molecules patterns (PAMPs) [131]. PRRs are expressed in most cells including T lymphocytes, B-cells, dendritic cells, macrophages, and epithelial cells [131,132]. Cellular signaling pathways and transcription factors that regulate inflammation and immune responses are activated by PAMPs and PRRs [131,132]. HIV-1 encoded TLR7 ligands enhance the production of the antiviral factor interferon-α (IFN-α) more robustly in females compared to males [133]. Also, 17β-estradiol enhances the production of TLR-7/9-mediated interferon-α responses in post-menopause women [48,49,134]. However, 17β-estradiol can suppress PAMPs’ responses and restrict inflammation [135–138]. Additionally, 17β-estradiol can abrogate NLRP3-mediated airway inflammation in asthma [139].

Levels of 17β-estradiol’s are higher in women during reproduction and pre-menopause age [140], and this correlates with robust innate immune responses and enhanced ability to clear viruses [52,141]. Lower levels of 17β-estradiol are observed during menopause and this corresponds to decreased immune responses and increased levels of the pro-inflammatory cytokines IL-6, IL-1β, and TNF-α [73,140]. The involvement of 17β-estradiol in these responses is supported by findings that 17β-estradiol supplementation suppresses the production of pro-inflammatory cytokines and boosts immune responses [47,73,142–144]. In contrast, high testosterone levels can reduce immune responses in response of the influenza vaccine [46,128]. Women as well as men with lower levels of testosterone both exhibit higher immune responses and more protection against infections [46]. Although different mechanisms are involved, 17β-estradiol suppresses infection of multiple viruses including influenza [51], HCV [80], Rubella virus [79], HIV-1 [78,133], HSV-1 [141], and SARS-CoV [54]. Experimentally in mice, greater levels of SARS-CoV infection were found in male mice [54] by a mechanism involving estrogen receptor-associated signaling [54], higher accumulation of inflammatory cells, and increased levels of some specific cytokines [IL-6,TNF-α,IL-1β], and chemokines (CXCL-1, CCL2). SARS-CoV2 induces an inflammatory storm in patients with severe symptoms [145], and multiple anti-inflammatory strategies are being tested for their ability to suppress virus-induced severe acute respiratory distress syndromes, including tocilizumab and anti-TNF-α therapy.

IL-6 and TNF-α both play important roles in inflammatory storms and ARDS development [146,147]. Tocilizumab, a humanized anti-human IL-6 receptor monoclonal antibody, has been used against COVID-19 and results show improvement in clinical symptoms and suppression of IL-6-mediated inflammation [148,149]. Similarly, anti-TNF therapies are being tested clinically to protect people at high risk for COVID-19 [149,150]. The anti-inflammatory effects of 17β-estradiol [73,74,151–153] include its ability to decrease ARDS by reducing inflammation and infiltration of immune cells in lungs [154], suppress LPS and burn trauma-induced acute lung injury [61], and attenuate NF-kB-mediated inflammation [63,155]. These might help explain results that women have fewer fatalities in COVID-19 and similarly with SARS-CoV and MERS-CoV.

## Upregulation of Endolysosomal Degradation Pathway by 17β-estradiol

Endolysosomes are acidic organelles that participate in the degradation of intracellular and extracellular macromolecules, components of plasma membranes, and cellular fragments [156–159]. In addition to their role in regulating autophagy, endolysosomes help regulate various cellular processes including membrane resealing, cell death, antigen presentation, cellular trafficking, and cell division [160–164]. Functional and structural changes to endolysosomes have been implicated in multiple diseases including cancer, neurodegenerative diseases, and infections [165–168].

Testosterone and 17β-estradiol both affect endolysosomes as well as the process of autophagy [95,169–173]. Testosterone upregulates expression levels of androgen-binding protein (ABP) [171] and androgens inhibit autophagy [172,174]. Moreover, testosterone enhances muscle mass by suppressing autophagy via AMPK inactivation [173]. In contrast, 17β-estradiol upregulates autophagy by diverse mechanisms [169]; it enhances lysosomal catabolic activity [170], promotes phagocytosis [175], and regulates lysosomal activity and autophagy by activating AMPK [169,176]. AMPK regulates RAAS; it enhances the phosphorylation and stability of ACE2 and controls endothelial homeostasis and pulmonary hypertension [177,178]. Additionally, the AMPK-p-ACE2 axis is impaired in human lungs with idiopathic pulmonary arterial hypertension (IPAH) [178]. Therefore, in COVID-19 patients, 17β-estradiol through increasing AMPK activity may up-regulate ACE2 and thereby suppress the development of severe symptoms [176,179–182].

Viral infection typically requires the involvement of endolysosomes [183,184] and SARS-CoV-2 is endocytosed following fusion with cell membranes in a pH-dependent manner [5,185]. Unclear, however, are mechanisms by which endocytosed virus is released from endolysosomes. Regardless, 17β-estradiol may stimulate endolysosomes to promote the degradation of cellular or extracellular materials [186]. Recently, it has been proved that SARS-CoV-2 blocks the autophagy pathway; however, spermidine reduce SARS-COV-2 infection by alleviating the lysosomal degradation pathway [187]. Hence, it needs to validate further that 17β-estradiol may restrict the SARS-CoV-2 infection by promoting the endolysosomal degradation pathway?

## Summary

SARS-CoV-2 infects men and women at the same rate, but men have a higher risk of developing severe complications and death. SARS-CoV-2 infects men and women at the same rate, but men have a higher risk of developing severe complications and death. Immune-compromised patients with hypertension, diabetes, cancer, and HIV-1 are at higher risk of being infected with the virus and developing severe symptoms including ARDS and death. 17β-estradiol might decrease SARS-CoV-2 infection by controlling RAAS, suppressing inflammatory storms, inducing anti-viral immune responses, and enhancing the virus’ degradation in endolysosomes by promoting the fusion of endosomes and lysosomes. High-risk patients may benefit from strategies designed to increase levels of 17β-estradiol by consuming estrogen pills and 17β-estradiol-enriched herbs [188,189].

Finally, it is important to address albeit briefly why postmenopausal women who have low levels of estradiol are still exhibiting lower death rates than are men from COVID-19. One reason might be the presence of catalytically-active mature natural killer (NK) cells (CD56dim); these cells are more plentiful in women than in men at ages greater than age 70 [37,190] and these cells may participate in suppression of SARS-CoV-2 infection. Other possible mechanisms may too be involved in protection of women from COVID-19.

## Figures and Tables

**Figure 1:**
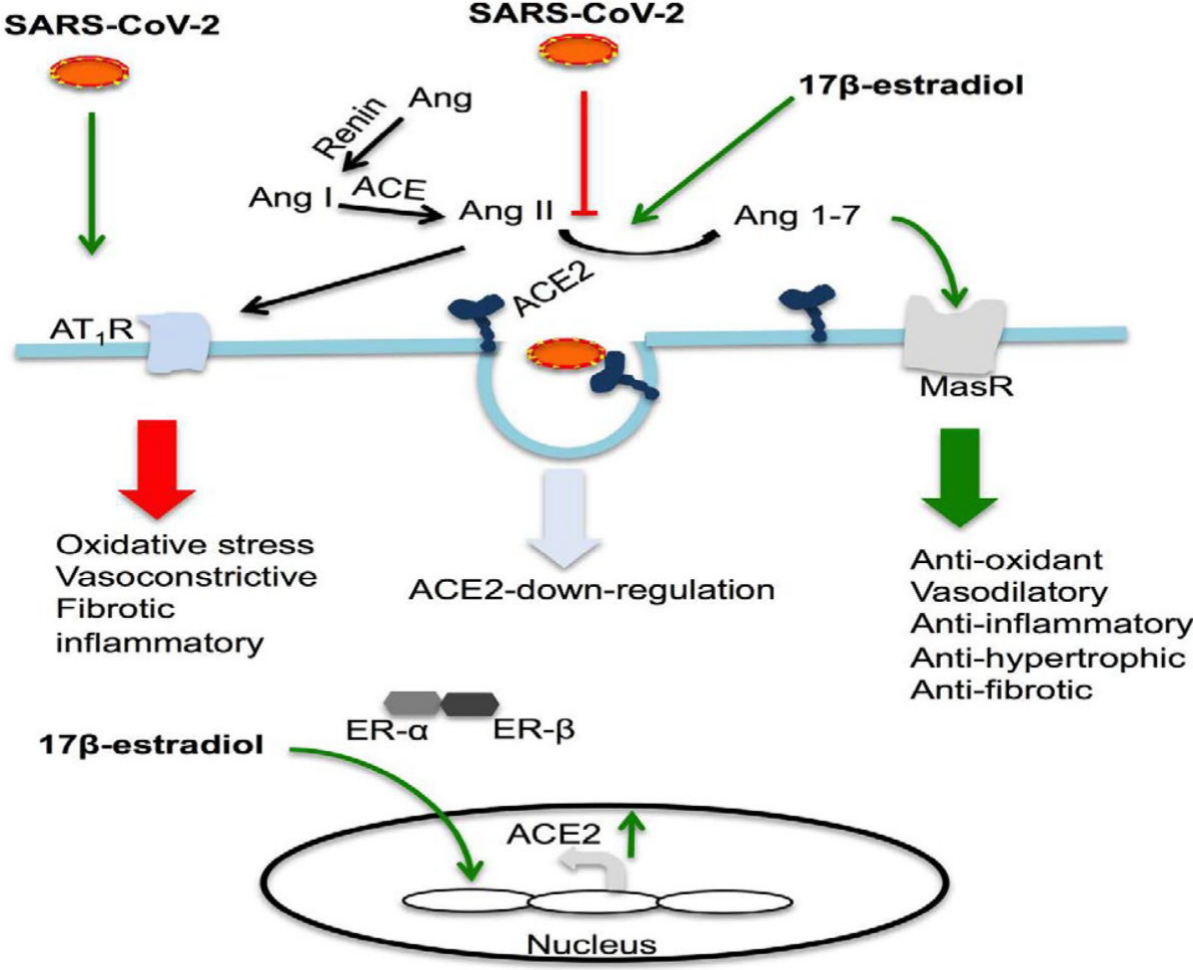
17β-estradiol regulates the RAAS system by enhancing ACE2 expression. SARS-CoV-2 may activate the ACE/AT1 axis by down-regulating ACE2 and thereby promoting the development of ARDS by inducing an inflammatory storm and increasing oxidative stress. 17β-estradiol may enhance expression levels of ACE2 (ACE2/Mas axis) and reduce ARDS. Activation of estrogen receptors regulates 17β-estradiol-mediated cellular signaling and gene expression. ACE catalyzes the conversion of Ang I to Ang II, which activates AT1 receptors. ACE2 catalyzes the conversion of Ang II to Ang I-7, which activates the Mas receptor. (SARS-CoV-2, severe acute respiratory syndrome-coronavirus 2; ER-α, estrogen receptor-α; ER-β, estrogen receptor-β; AT1R, angiotensin II type 1 (AT1) receptor; ACE, angiotensin-converting enzyme; ACE2, angiotensin-converting enzyme 2; MasR, Mas receptor; Ang II, angiotensin II; Ang 1–7, angiotensin 1–7).

**Figure 2:**
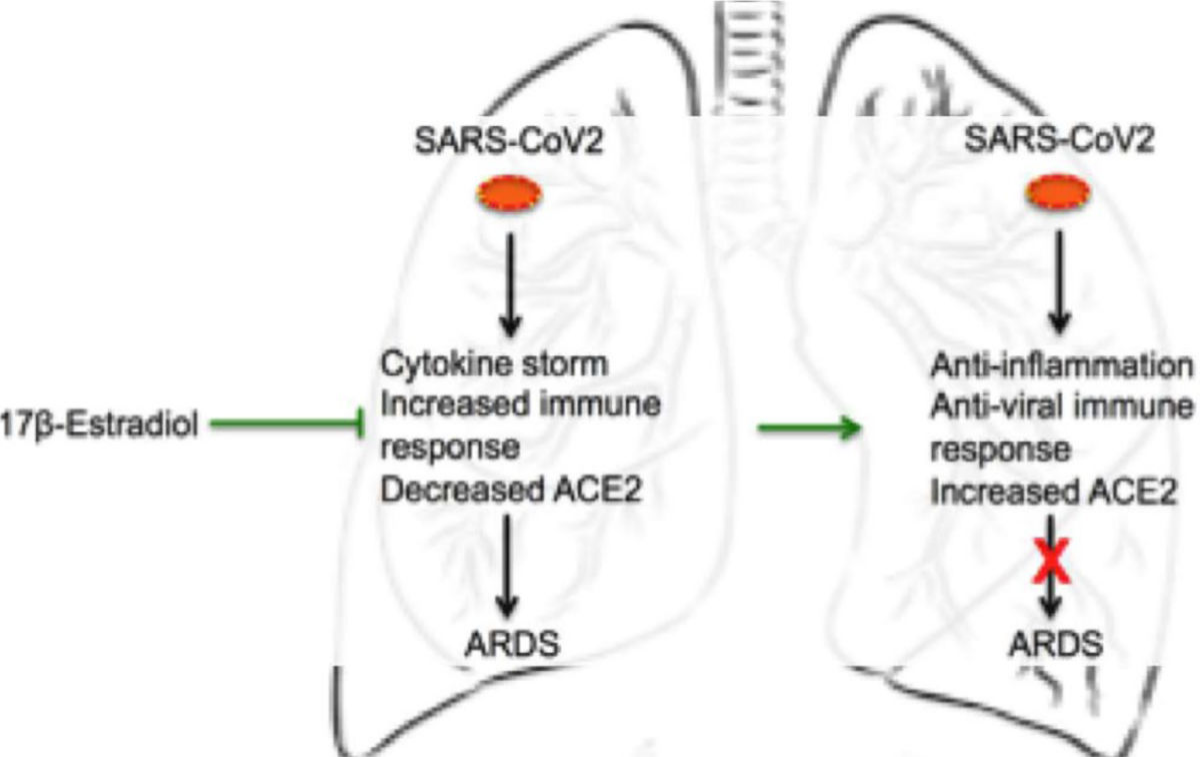
17β-estradiol might suppress ARDS by enhancing anti-viral and anti-inflammatory immune responses. SARS-CoV-2 develops severe complications in infected people including ARDS, by the down-regulating ACE2 expression and inducing a massive inflammatory storm. However, 17β-estradiol might suppress ARDS by, for example, controlling the RAAS system, and enhancing anti-inflammatory and anti-viral immune responses. (SARS-CoV-2: Severe Acute Respiratory Syndrome-Coronavirus-2; ACE2: Angiotensin-Converting Enzyme; ARDS: Acute Respiratory Distress Syndrome).

**Figure 3:**
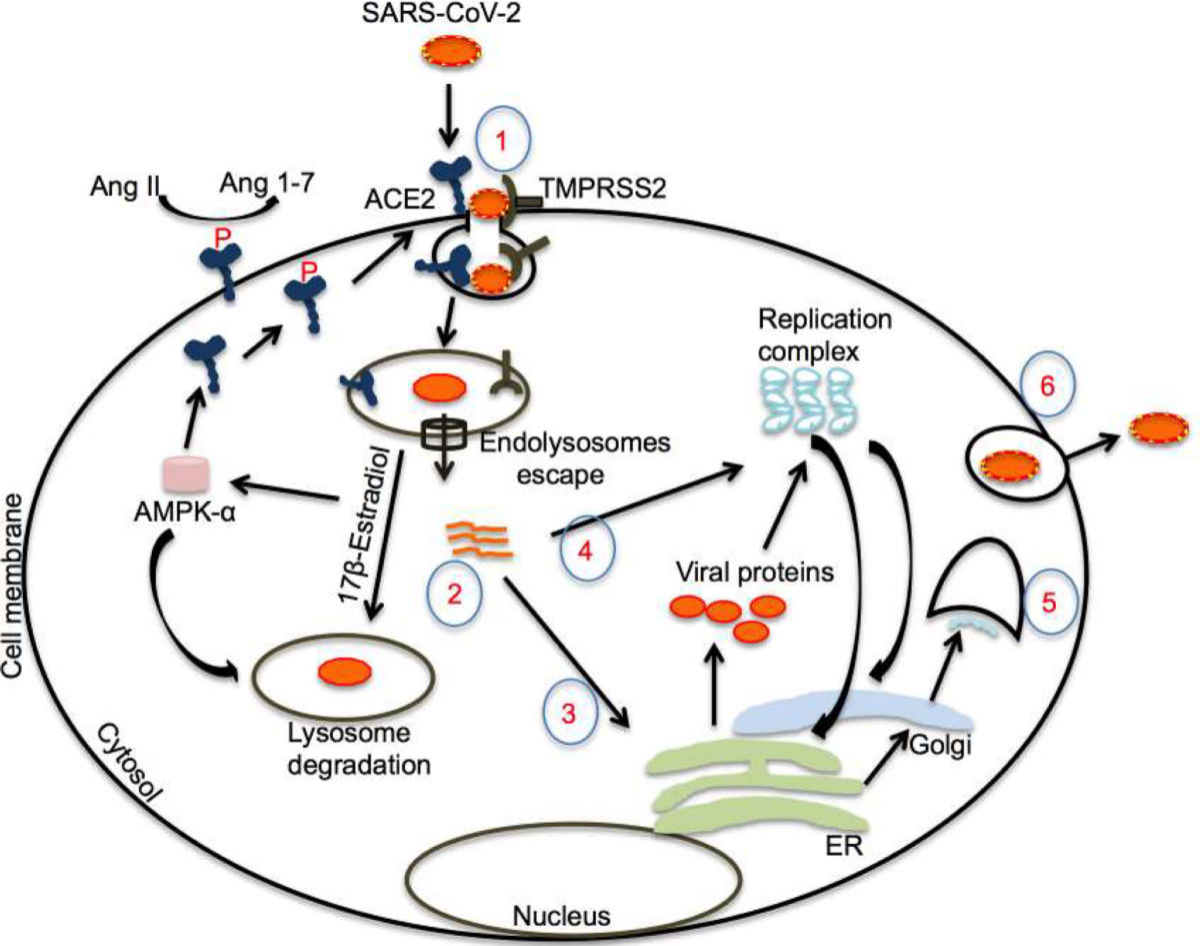
Upregulation of endolysosomal degradation pathway by 17β-estradiol. The virus infects the cell by binding spike proteins with ACE2 on cell membranes following priming by TMPRSS2. Following endolysosome escape, RNA can accumulate in the cytosol, where it participates in protein translation. Translated proteins produce a replication complex to make viral RNA. 17β-estradiol may enhance the endolysosome’s degradation of the virus by enhancing the fusion of endosomes and lysosomes, probably by increasing AMPK activity and other possible mechanisms. Moreover, 17β-estradiol-mediated AMPK activation may enhance ACE2 stability by inducing phosphorylation and reduce ARDS and pulmonary hypertension. (SARS-CoV2: Severe Acute Respiratory Syndrome-Coronavirus 2; ACE2: Angiotensin-Converting Enzyme 2; p-ACE2: Phosphor-Angiotensin-Converting Enzyme 2; TMPRSS2: Transmembrane Protease, Serine 2; Ang II: Angiotensin II; Ang 1–7: Angiotensin 1–7; AMPKα: Adenosine Monophosphate Kinase-α; ER: Endoplasmic Reticulum).
